# An Essential Role for DYF-11/MIP-T3 in Assembling Functional Intraflagellar Transport Complexes

**DOI:** 10.1371/journal.pgen.1000044

**Published:** 2008-03-28

**Authors:** Chunmei Li, Peter N. Inglis, Carmen C. Leitch, Evgeni Efimenko, Norann A. Zaghloul, Calvin A. Mok, Erica E. Davis, Nathan J. Bialas, Michael P. Healey, Elise Héon, Mei Zhen, Peter Swoboda, Nicholas Katsanis, Michel R. Leroux

**Affiliations:** 1 Department of Molecular Biology and Biochemistry, Simon Fraser University, Burnaby, British Columbia, Canada; 2 McKusick-Nathans Institute of Genetic Medicine, Johns Hopkins University School of Medicine, Baltimore, Maryland, United States of America; 3 Karolinska Institute, Department of Biosciences and Nutrition, Södertörn University College, School of Life Sciences, Huddinge, Sweden; 4 Samuel Lunenfeld Research Institute, Mount Sinai Hospital and Department of Microbiology and Medical Genetics, University of Toronto, Ontario, Canada; 5 Department of Ophthalmology and Vision Sciences, The Hospital for Sick Children and University of Toronto, Toronto, Ontario, Canada; Washington University, United States of America

## Abstract

MIP-T3 is a human protein found previously to associate with microtubules and the kinesin-interacting neuronal protein DISC1 (Disrupted-in-Schizophrenia 1), but whose cellular function(s) remains unknown. Here we demonstrate that the *C. elegans* MIP-T3 ortholog DYF-11 is an intraflagellar transport (IFT) protein that plays a critical role in assembling functional kinesin motor-IFT particle complexes. We have cloned a loss of function *dyf-11* mutant in which several key components of the IFT machinery, including Kinesin-II, as well as IFT subcomplex A and B proteins, fail to enter ciliary axonemes and/or mislocalize, resulting in compromised ciliary structures and sensory functions, and abnormal lipid accumulation. Analyses in different mutant backgrounds further suggest that DYF-11 functions as a novel component of IFT subcomplex B. Consistent with an evolutionarily conserved cilia-associated role, mammalian MIP-T3 localizes to basal bodies and cilia, and zebrafish *mipt3* functions synergistically with the Bardet-Biedl syndrome protein Bbs4 to ensure proper gastrulation, a key cilium- and basal body-dependent developmental process. Our findings therefore implicate MIP-T3 in a previously unknown but critical role in cilium biogenesis and further highlight the emerging role of this organelle in vertebrate development.

## Author Summary

The transport of protein complexes and associated cargo along microtubule tracks represents an essential eukaryotic process responsible for a multitude of cellular functions, including cell division, vesicle movement to membranes, and trafficking along dendrites, axons, and cilia. The latter organelles are hair-like cellular appendages implicated in cell and fluid motility, sensing and transducing information from their environment, and development. Their biogenesis and maintenance depends on a kinesin- and dynein-mediated motility process termed intraflagellar transport (IFT). In addition to comprising these specialized molecular motors, the IFT machinery consists of large multisubunit complexes whose exact composition and organization has not been fully defined. Here we identify a protein, DYF-11/MIP-T3, that is conserved in all ciliated organisms and is associated with IFT in *C. elegans*. Disruption of *C. elegans* DYF-11 results in structurally compromised cilia, likely as a result of IFT motor and subunit misassembly. Animals lacking DYF-11 display chemosensory anomalies, consistent with a role for the protein in cilia-associated sensory processes. In zebrafish, MIP-T3 is essential for gastrulation movements during development, similar to that observed for other ciliary components, including Bardet-Biedl syndrome proteins. In conclusion, we have identified a novel IFT machinery component that is also essential for development in vertebrates.

## Introduction

Cilia are slender subcellular structures that protrude from the surfaces of most eukaryotic cell types, where they carry out functions associated with sensation and/or motility. Motile cilia are used for the locomotion of spermatozoa or organisms such as the unicellular green alga *Chlamydomonas reinhardtii*, as well as for generating fluid flow, as is the case in respiratory airways [1]. Non-motile (primary) cilia are nearly ubiquitous in multicellular organisms, and perform a wide range of sensory functions, including chemosensation/olfaction, photoreception, and mechanosensation [1]–[5]. Primary cilia are also associated with several signaling processes critical for development, including Hedgehog signaling, PDGFRαα signaling, as well as canonical and non-canonical (planar cell polarity) Wnt signaling pathways [6]–[12]. Hence, defects in ciliary structure or function affect nearly every organ in humans, and are associated with several pleiotropic genetic disorders. For example, Bardet-Biedl syndrome (BBS), Alström syndrome, Meckel syndrome, Senior-Løken syndrome, Joubert syndrome, and several cystic kidney diseases are all believed to involve dysfunction of primary cilia and/or basal bodies, the modified centriolar structures that nucleate ciliary axonemes [13]–[20].

Cilia are organelles that require several hundred proteins to support their motility and/or sensory and signaling functions [21],[22]. Of particular relevance to the present study, cilia possess a specialized microtubule-based transport system, termed intraflagellar transport (IFT), which shuttles IFT complexes bi-directionally along the axoneme and supports the formation and maintenance of the organelles [23]–[25]. The IFT particles, first observed in *Chlamydomonas* [26] consist of anterograde Kinesin-2 motor(s) that move cargo into the cilia and a retrograde dynein motor involved in recycling components back to the base (basal body). The molecular motors are associated with two biochemically-separable multisubunit assemblies termed IFT particle subcomplexes A and B [27]–[29]. In *Chlamydomonas*, IFT subcomplexes A and B consist of at least 6 and 11 subunits, respectively.

In recent years, the nematode *Caenorhabditis elegans* has emerged as a powerful model organism for the study of cilia and ciliogenesis. The cilia of *C. elegans* are non-motile and restricted to a subset of sensory neuronal cells principally localized in the head and tail of the animal [30]. While structurally similar to the canonical flagella of *Chlamydomonas*, *C. elegans* cilia emanate from a potentially more degenerate basal body (termed transition zone) and exhibit, following the doublet microtubule-containing ciliary middle segment, a pronounced extension of singlet axonemal microtubules in their distal segments [30]. Despite these differences, most, if not all, of the core IFT components identified in *Chlamydomonas* appear to be conserved in nematodes [31]. Additionally, several other proteins associated with and necessary for the function of the IFT machinery and cilium formation have been discovered in *C. elegans*, namely DYF-1 [32] DYF-2 [33], DYF-3 [34], DYF-13 [35], and IFTA-1 [36]. Like known IFT particle subcomplex A/B components, orthologs of these *C. elegans* proteins are enriched within the membrane-plus-matrix fraction of the recently identified *Chlamydomonas* flagellar proteome [37], supporting the notion that they represent conserved IFT components. In addition, research in the worm has shown that Bardet-Biedl Syndrome (BBS) proteins are themselves associated with IFT and are required to maintain the integrity of the IFT particle during transport along the cilium [4],[32]. The discovery of novel *C. elegans* IFT proteins suggests that the IFT machinery is more complex than suspected originally from biochemical fractionation studies, raising the possibility that additional components critical for IFT have yet to be identified.

On the basis of several bioinformatic, genomic and proteomic studies, we recently surmised [21] that the microtubule-associated MIP-T3/TRAF3IP1 protein likely represents a previously unknown but conserved ciliary protein. Here, we show that the *C. elegans dyf-11* mutant harbors a loss of function mutation in the gene encoding the MIP-T3 ortholog. We demonstrate that the *dyf-11* gene product, DYF-11, is a novel IFT-associated protein required for the proper assembly and function of the IFT machinery, as well as the sensory abilities of cilia. Consistent with these findings, mammalian MIP-T3 localizes to the basal body in a pre-ciliated cell and to the ciliary axoneme in ciliated cells. Furthermore, suppressing the *Danio rerio* (zebrafish) *mipt3* gene leads to a range of developmental defects recently associated with cilia function, most prominently cell movement anomalies during gastrulation, a process under the control of the non-canonical Wnt signaling pathway [12]. Our findings therefore demonstrate new roles for the evolutionarily conserved DYF-11/MIP-T3 protein in building and maintaining functional cilia, and in developmental signaling pathways.

## Results/Discussion

### The *C. elegans* MIP-T3 Gene Ortholog C02H7.1 Is Disrupted in *dyf-11* Mutants

**Figure 1:**
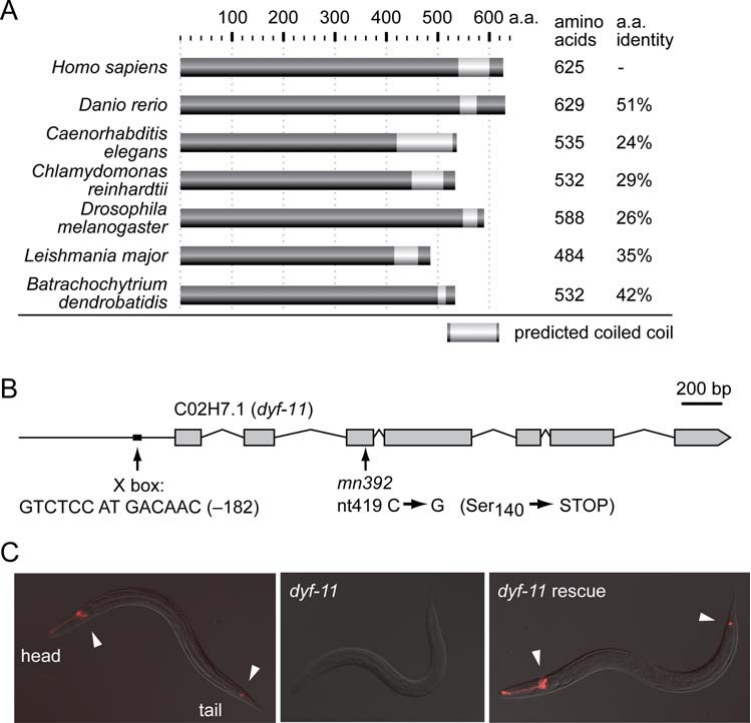
The *C. elegans dyf-11* strain contains a mutation in the gene C02H7.1, the MIP-T3 ortholog. (A) Predicted protein structure of *C. elegans* C02H7.1/DYF-11 as compared to MIP-T3 orthologs in *H. sapiens* (human; AAF76984), *D. rerio* (zebrafish; Q6PGZ3), *C. reinhardtii* (green alga; C_140070), *D. melanogaster* (fruit fly; CG3259), *L. major* (a kinetoplastid parasite; CAJ04965), and the ciliated fungus *B. dendrobatidis* (BDEG_02985.1). (B) Cloning of *dyf-11(mn392)* was accomplished by identifying a nonsense mutation in the third exon of the *C. elegans* C02H7.1 gene. Note the presence of an X box at nucleotide -182 bp relative to the ATG start codon. Exons are denoted with gray boxes and intervening introns with lines. (C) Dye-filling assays with the red fluorescent dye, DiI, reveal that compared to wild-type (N2) animals, *dyf-11(mn392)* mutants do not visibly take up dye (a Dyf phenotype). A transgene expressing wild-type C02H7.1 fused to GFP in *dyf-11* animals rescues this defect. Fluorescence microscopy signals (all 100 msec exposure) are overlaid onto DIC images. Arrowheads indicate positions of dye-filling neuronal cell bodies.

Several lines of evidence support the notion that MIP-T3 orthologs have a ciliary function [21]. First, MIP-T3 is found exclusively in ciliated organisms [38],[39]. Second, its expression in *C. elegans* and *Drosophila* is restricted to ciliated cells and under the control of an X box motif, which regulates genes required for ciliogenesis [35],[38],[40],[41]. Similarly, *Chlamydomonas* MIP-T3 (C_140070) is upregulated during flagellar regeneration, and proteomic analyses uncovered MIP-T3 in the *Chlamydomonas* flagellar proteome [37],[42]. MIP-T3 proteins range in size from 484 to 625 amino acids and have no recognizable domains except for a predicted coiled-coil region near the C-terminus (Figure 1A), providing no indication of their specific cellular function(s).

To test the hypothesis that MIP-T3 encodes a ciliary protein and to analyze its *in vivo* function, we sought to obtain a strain with a disruption in the *C. elegans* C02H7.1 open reading frame that encodes MIP-T3 (Figure S1). We noticed that a previously identified mutant, *dyf-11(mn392)*, whose compromised fluorescent dye uptake is suggestive of cilia dysfunction [43], maps within a genetic interval that contains C02H7.1. Sequencing of C02H7.1 in the *dyf-11(mn392)* mutant strain revealed a nonsense mutation predicted to give rise to a null allele (Figure 1B). Importantly, the dye-filling defect of *dyf-11(mn392)* is fully rescued by introducing a transgene expressing the wild-type copy of the C02H7.1 coding region fused to GFP (Figure 1C). This confirms the cloning of *dyf-11* and demonstrates the functional nature of the GFP-tagged C02H7.1 protein, which we use below for *in vivo* characterization. We henceforth refer to the *C. elegans* MIP-T3 gene ortholog C02H7.1 as *dyf-11*, and its gene product as DYF-11.

### DYF-11 Is Required for the Formation of Structurally Intact and Functional Cilia

**Figure 2 pgen-1000044-g002:**
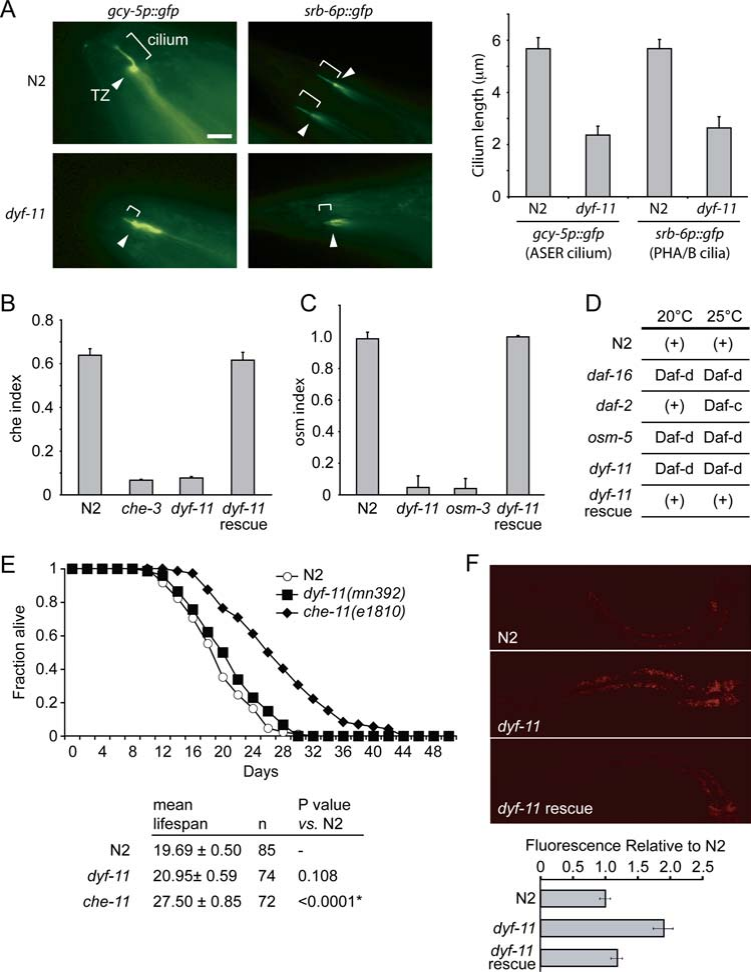
*dyf-11* (*mn392*) mutants display ciliary structure defects, behavioural phenotypes indicative of ciliary anomalies, and a lipid accumulation phenotype. (A) Cilium length measurements using the *gcy-5p::gfp* and *srb-6p::gfp* transcriptional markers (which highlight the cell bodies, axons, dendrites, transition zones/basal bodies and cilia of the ASER and phasmid neurons with GFP, respectively) reveal that the *dyf-11(mn392)* strain has truncated cilia relative to wild-type (N2) animals. Measurements (in µm), shown in the graph, are from the transition zone (TZ, arrowhead) to the tip of the cilium. Scale bar, 5 µm. (B, C) *dyf-11* mutants are defective in both chemotaxis and osmo-avoidance, as observed from their significantly lower che or osm indexes compared to wild-type (N2) animals, respectively. The *dyf-11* mutant strain defects are comparable to those of other cilia mutants (*che-3* and *osm-3*) and can be rescued by transgenic expression of wild-type DYF-11::GFP. (D) *dyf-11* mutants are dauer formation defective (Daf-d) at 20°C and 25°C. The ciliary (*osm-5*) or insulin signaling (*daf-2* and *daf-16*) mutants are Daf-d or Daf-c (constitutively enter the dauer stage) at the indicated temperatures. (+), ability to enter the alternate dauer stage. (E) Lifespan analyses show that *dyf-11* mutants have statistically normal longevity when compared to wild-type (N2) animals. *che-11* ciliary mutants, on the other hand, are significantly longer lived. Mean lifespan, number of worms assayed, and P value compared to N2 are tabulated. (F) *dyf-11* mutants display an increase in lipid content (relative to N2 animals) that is rescued by the wild-type DYF-11::GFP transgene. Lipid content is assessed by Nile Red staining followed by quantitation of relative fluorescence intensities.

To determine if the dye-filling anomaly observed in the *dyf-11* strain stems from structural defects in cilia that are normally exposed to the environment, we expressed GFP in the ASER amphid (head) or two PHA/B phasmid (tail) neurons using reporter constructs driven by the *gcy-5* or *srb-6* gene promoters, respectively; in those neurons, GFP diffuses freely to highlight the entire cell, including the cell body, axon, dendrite, transition zone/basal body and cilium, so as to permit cilium length measurements [44]. Although no morphological defects with the dendritic processes or transition zone positioning were observed, the cilia of *dyf-11* mutants were truncated substantially (2.4±0.3 µm and 2.6±0.4 µm for amphids and phasmids, respectively) compared to those of wild-type animals (both 5.7±0.4 µm) (Figure 2A). These findings likely explain the dye-filling defect in *dyf-11* animals and directly implicate MIP-T3 in ciliogenesis. Additionally, the observed lengths of *dyf-11* mutant cilia are similar to those of IFT subcomplex B mutants.

We next used established assays [30], [45]–[47] to ask whether *dyf-11* animals exhibit phenotypes consistent with cilia dysfunction, including anomalies in chemotaxis, avoidance of high osmolarity, ability to form stress-resistant dauer larvae, and lifespan.

We first compared the ability of wild-type and *dyf-11* mutant animals to detect a volatile odorant, isoamyl-alcohol. Although the *dyf-11* mutants are not impaired in their movement (data not shown), they show a pronounced inability to chemotax towards the attractant, indicative of an abnormal Chemotaxis (Che) phenotype (Figure 2B). In addition, compared to their wild-type counterparts, *dyf-11* animals show a clear defect in avoiding moving into a solution of high osmolarity (8 M glycerol; Figure 2C), an Osmotic avoidance-abnormal (Osm) phenotype frequently encountered in ciliary mutants [30],[45]. These *dyf-*11 mutant phenotypes are consistent with the original observations of Starich *et al.* [43].

We then tested for the ability of *dyf-11* mutants to enter the alternative dauer lifestage at two different temperatures (20/25°C). While wild-type larvae become dauers at these temperatures upon starvation, mutants with defects in IFT (*e.g.*, *osm-5*) or the cilium-dependent insulin signaling pathway (*e.g.*, *daf-16* and *daf-2*) are either dauer formation-defective (Daf-d) or constitutively form dauer larvae (Daf-c) [47] (Figure 2D). We found that *dyf-11* mutants are Daf-d at both temperatures, consistent with abrogated ciliary function [30],[43],[47] (Figure 2D). Lastly, we noted severe male mating defects (data not shown), which can probably be ascribed to the improper mechanosensory/chemosensory functions of cilia in the male tail [48]. For all four behavioral phenotypes observed, the defects could be rescued by expression of the GFP-tagged wild-type DYF-11 protein (Figures 2B–D and data not shown for male mating). Finally, although some ciliary mutants are long-lived [49], for example the *che-11* mutant, we found no statistical difference in lifespan between wild-type and *dyf-11* animals (Figure 2E).

Intriguingly, *dyf-11* mutants also show, compared to the wild-type and rescue strains, a pronounced increase in Nile Red staining within intestinal cells, indicative of an increased lipid accumulation phenotype (Figure 2F). This result is remarkable, as past *C. elegans* screens for lipid accumulation identified only two ciliary proteins, namely the *tubby* obesity-associated gene ortholog TUB-1 [50] and BBS-1, which is linked to the obesity disorder Bardet-Biedl syndrome [51]. These observations, coupled with our present findings with *dyf-11*, suggest an evolutionarily-conserved connection between cilia and lipid homeostasis. It should be noted that since the genome-wide lipid accumulation screens were RNAi-based, it is likely that many more ciliary proteins are involved in lipid homeostasis, as RNAi has been found to be considerably less penetrant in ciliated neurons.

Altogether, our cilium length measurements and sensory behavioral analyses (Che, Osm and Daf) demonstrate that the *dyf-11* mutant strain possesses prominent structural and functional ciliary defects, confirming our hypothesis that MIP-T3 plays a role in ciliogenesis and cilia function. The role(s) of DYF-11/MIP-T3 in cilia formation and function appear to be highly specific, as we have not noted any gross morphological, developmental or locomotory defects in *dyf-11* mutant animals.

### DYF-11/MIP-T3 is a Novel Intraflagellar Transport (IFT) Protein

**Figure 3:**
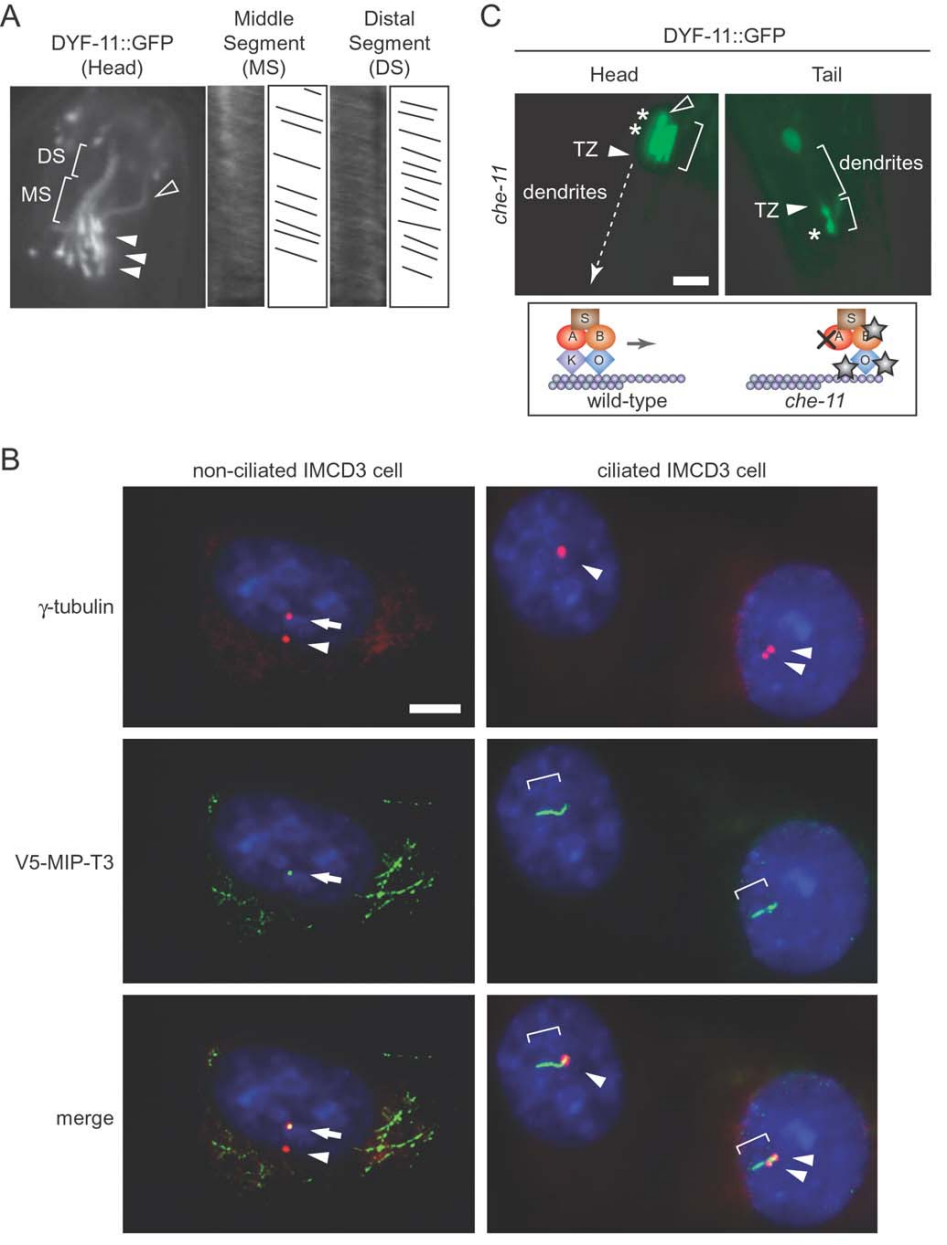
DYF-11 is a component of the intraflagellar (IFT) transport machinery. (A) Kymograph analyses show the bi-phasic anterograde movement of DYF-11::GFP in amphid cilia. Middle segment (MS) motion was determined to be 0.74±0.08 µm/sec, while the distal segment (DS) velocity was 1.15±0.21 µm/sec. The second and fourth panels are actual kymographs, whereas the third and fifth panels are representative traces of moving particles in the indicated middle or distal segments; X axes denote time whereas Y axes denote distance covered by fluorescently-labeled particles. In the still fluorescence microscopy image taken from Movie S1 (left panel), solid arrowheads denote the positions of representative transition zones, while hollow arrowheads denote unexpected dendritic endings. (B) Human MIP-T3 tagged with a V5 epitope (V5-MIP-T3) localizes to centrosomes in IMCD3 kidney cells that have not yet ciliated, and to the axoneme when cilia are present. Centrosomes/basal bodies are stained with an antibody against γ-tubulin (red), V5-MIP-T3 is marked with an antibody against the V5 epitope (green), and the nucleus is stained with DAPI (blue). Arrowheads and arrows point to centrosomes/basal bodies, and brackets denote ciliary axonemes. Scale bar, 5 µm. (C) Similar to other well characterized IFT components, DYF-11::GFP accumulates in the cilia of *che-11* mutants, which displays retrograde transport defects. Arrowheads represent the positions of transition zones, brackets show ciliary axonemes, and stars denote accumulations (compare the localization of DYF-11::GFP in head amphid cilia of wild-type (A) and *che-11* mutant (C) animals; see also Figure 4A for normal DYF-11::GFP localization). Scale bar, 5 µm. Schematics represent anterograde transport in wild-type animals (no accumulations) and *che-11* mutants (accumulations at the tip of cilia, shown as stars). K, Kinesin-II; O, OSM-3-kinesin; A, IFT subcomplex A; B, IFT subcomplex B; S, BBS protein complex. TZ, transition zones.

**Figure 4:**
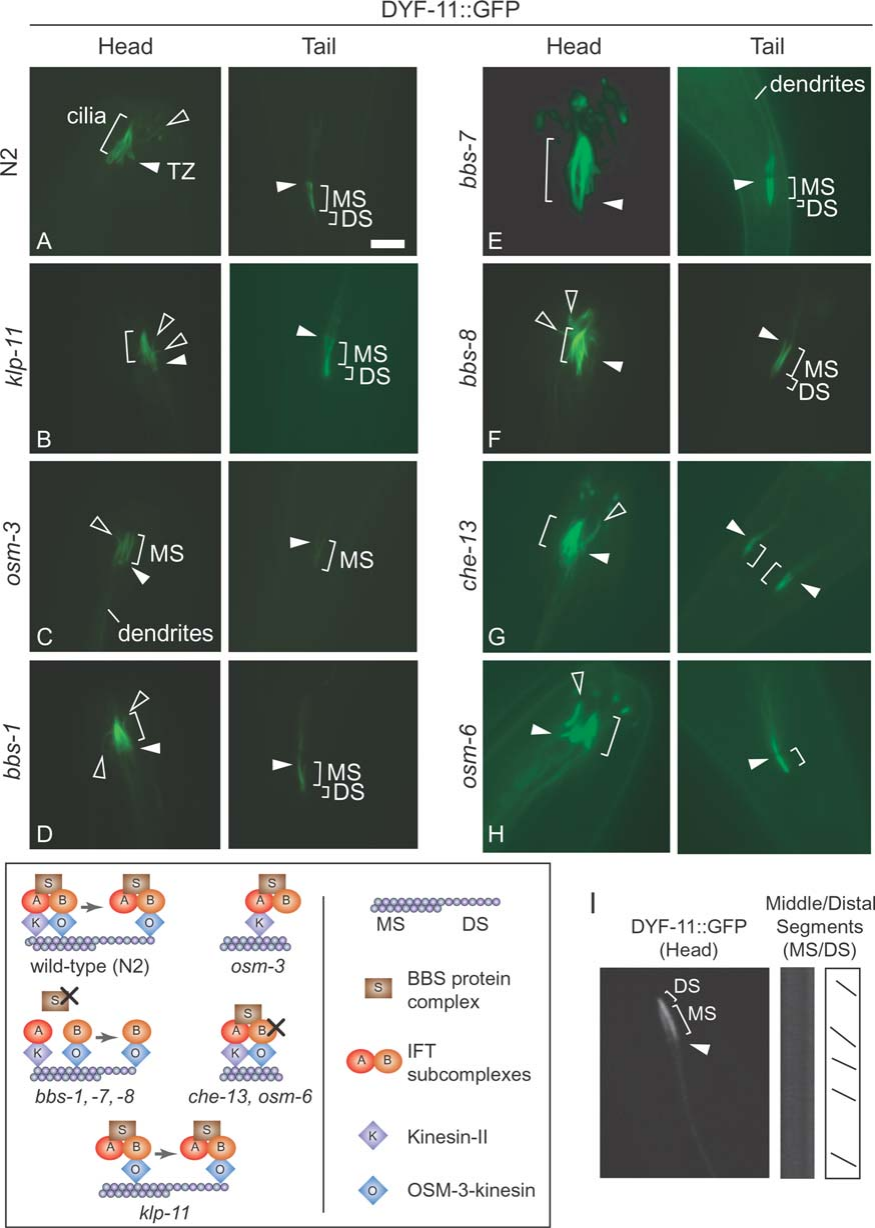
*C. elegans* DYF-11::GFP may be functionally associated with IFT particle subcomplex B. (A, B) DYF-11::GFP localizes along the entire length of amphid/phasmid cilia in wild-type (N2) and *klp-11* (a motor component of heterotrimeric Kinesin-2) animals, both of which have full-length cilia. Note the schematics at the bottom of the figure. (C) DYF-11::GFP is observed along the length of the middle segments of *osm-3* (homodimeric Kinesin-2) mutants, which lack the distal segments (see schematic). (D-F) DYF-11::GFP localizes along the entire middle segments and truncated distal segments of *bbs* mutant worms, consistent with the hypothesis that DYF-11 is associated with OSM-3-kinesin/IFT subcomplex B (Kinesin-II/subcomplex A does not enter the distal segment; see schematic). Middle segments (MS) and distal segments (DS) are specifically highlighted. (G, H) DYF-11::GFP ciliary localization is not perturbed in the truncated cilia of two subcomplex B mutants (*che-13* and *osm-6*, respectively; see schematic). Transition zones (TZ) are shown by white arrowheads, unusual dendritic termini are denoted by hollow arrowheads, and brackets designate the observed ciliary axonemes. Note that DYF-11 behaves like an IFT subcomplex B component (*i.e.*, moves into the cilia of *osm-3*, *klp-11* as well as distal segment of *bbs* mutants). Scale bar, 5 µm. (I) Kymograph analyses show the movement of DYF-11::GFP in a *bbs-7* mutant animal at a velocity of 1.21±0.19 µm/sec along the middle and distal segments of phasmid cilia, consistent with an association with OSM-3-kinesin.

To directly observe whether the *C. elegans* DYF-11 protein associates with ciliary structures (transition zones/basal bodies and/or cilia), we generated transgenic lines bearing a translational fusion construct of the complete *dyf-11* gene (with its endogenous promoter) and GFP. Fluorescence microscopy observation of the lines revealed that *dyf-11::gfp* is expressed specifically in ciliated (*e.g.*, amphid and phasmid) sensory neurons (data not shown), consistent with expression patterns obtained using transcriptional GFP-fusion constructs obtained in two large-scale studies [35],[52]. Importantly, the DYF-11::GFP protein was found to be highly enriched at transition zones and within ciliary axonemes (Figure 3A, Figure 4). This subcellular localization is indistinguishable from that of other *C. elegans* proteins associated with IFT, including BBS proteins, newly-discovered IFT proteins, and IFT particle subcomplex A and B proteins [32]–[36],[53]. Consistent with an evolutionarily-conserved role for MIP-T3 at basal bodies and cilia, V5 epitope-tagged human MIP-T3 also localizes to the basal body in cells that have not yet ciliated, and to the ciliary axonemes of ciliated IMCD3 kidney cells (Figure 3B).

Time-lapse microscopy in *C. elegans* revealed that DYF-11::GFP localization is not static; the GFP-tagged protein moves bi-directionally along the length of amphid and phasmid ciliary axonemes (Figure 3A; Movie S1). Kymograph analyses show that DYF-11::GFP anterograde movement is bi-phasic, exhibiting a velocity of 0.74±0.08 µm/sec along doublet microtubules in middle segments and 1.15±0.21 µm/sec along singlet microtubules in distal segments (Figure 3A). These observations show that DYF-11 is transported cooperatively by Kinesin-II and OSM-3-kinesin in middle segments and then OSM-3-kinesin alone in distal segments, exactly as with other *C. elegans* IFT/BBS proteins [32],[54]. Importantly, DYF-11::GFP motility depends on the IFT machinery itself, as abrogating the IFT particle subcomplex A protein CHE-11/IFT140, which is required for retrograde transport, results in the expected accumulation of DYF-11::GFP at distal tips (Figure 3C).

Interestingly, DYF-11::GFP can be observed in several additional dendritic extensions that are not typically seen with established GFP-tagged IFT protein markers (see hollow arrowheads in Figure 3A and Figure 4). At least some of these unexpected structures appear to overlap with the cilia of the wing neuron AWC in the DYF-11::GFP-containing strain (Figure S2), which might indicate that the other unaccounted-for structures represent the axonemes of the remaining wing neurons (AWA, AWB). This unexpected observation may reflect stronger accumulation of DYF-11::GFP in the wing neuron cilia, or could be indicative of additional roles for DYF-11 in the development of morphologically distinct cilia (wing cilia have elaborate structures) [30]. Another possible explanation for the observed dendritic extensions is that they represent elongated cilia not sufficiently aligned to enter the amphid channel, a phenotype reminiscent of those recently seen in *dyf-5* mutants, which have defects in IFT-kinesin-mediated transport [55].

### DYF-11 Is Transported in the Cilium in a Manner Similar to IFT Particle Subcomplex B

The molecular architecture of the motor-IFT machinery has been studied in some detail, using mainly *Chlamydomonas* and *C. elegans* as model systems [25], [28], [54], [56]–[60]. In *C. elegans*, the motor-IFT machinery consists of at least 32 components organized into three main modules [58]: a motor module with two kinesin-2-like anterograde motors for anterograde transport (the more canonical heterotrimeric Kinesin-II, and homodimeric OSM-3) and a dynein motor for retrograde transport; another module containing two IFT particle multisubunit subcomplexes (A and B) that are separable genetically and biochemically [27]–[29],[32],[54],[58]; finally, a BBS protein complex/module that mediates the association between Kinesin-II/subcomplex A and OSM-3/subcomplex B [4],[32],[61]. Having identified DYF-11 as a novel IFT protein, we sought to characterize its spatial relationship (*i.e.*, localization in one of the aforementioned modules) and function with respect to other components of the IFT machinery.

To test whether DYF-11 may be a component of either the Kinesin-II or OSM-3 anterograde motor modules, similar to the association of DYF-1 with OSM-3 [32], we queried whether GFP-tagged DYF-11 enters the ciliary axonemes of mutants lacking either motor (compared with Figure 4A, which shows DYF-11::GFP in wild-type animals). We found that in the *klp-11* mutant, which lacks Kinesin-II motor function, DYF-11::GFP readily entered the full-length cilia produced by the redundant OSM-3-kinesin (Figure 4B). Likewise, DYF-11::GFP could be detected along the entire length of the ciliary middle segments in the *osm-3* kinesin mutant, which specifically lacks distal segments (Figure 4C). These observations suggest that the assembly of DYF-11 with the motor-IFT particle machinery is not dependent on either of the kinesin anterograde motors, and leaves the possibility that it is more closely associated with the BBS protein complex or the IFT subcomplexes A or B.

We therefore analyzed the behavior of DYF-11::GFP in the three available *bbs* mutants (*bbs-1*, *bbs-7*/*osm-12* and *bbs-8*). In all *bbs* mutants, DYF-11::GFP was distributed throughout the middle segment and the residual distal segment (Figures 4D–F). This suggests that DYF-11 is not tightly associated with BBS protein(s), since all examined BBS proteins are unable to enter cilia in animals lacking *bbs-1*, *bbs-7*/*osm-12* or *bbs-8* [4],[32],[58]. This is also consistent with the fact that the *dyf-11* mutant cilia are distinctly shorter (Figure 2A) than those of *bbs* mutant cilia, which possess part of the distal segment [32],[51],[58]. Instead, the ability of DYF-11::GFP to enter the residual distal segment of *bbs* mutants implies that it is associated with the OSM-3-kinesin/IFT subcomplex B; this conclusion stems from the fact that in *bbs* mutants, all tested IFT subcomplex B components enter the distal segment in an OSM-3-dependent manner, whereas subcomplex A components are transported by Kinesin-II, and do not enter the distal segment [32] (see also schematics in Figure 4). Furthermore, DYF-11:GFP travels at a velocity of 1.21±0.19 µm/sec throughout the middle and distal segments of the cilia of *osm-12/bbs-7* mutant worms (Figure 4I), indicative of an OSM-3-dependent transport process typically seen for IFT subcomplex B components in a *bbs* mutant background [4],[32],[58].

**Figure 5:**
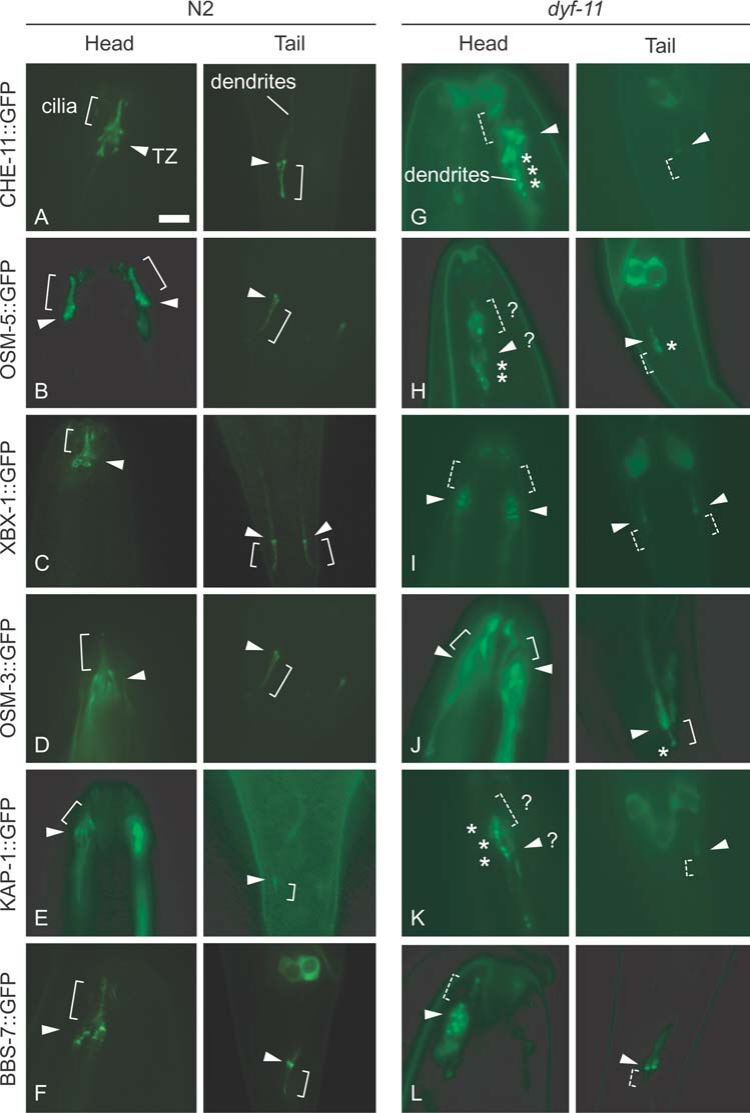
*C. elegans* DYF-11 is required for the assembly and function of many components of the motor-IFT machinery, including Kinesin-II. (A-F) In wild-type (N2) animals, 6 representative GFP-tagged IFT-associated proteins (CHE-11, OSM-5, XBX-1, OSM-3, KAP-1, and BBS-7/OSM-12) show the expected prominent localization to transition zones (TZ; arrowheads) and cilia (brackets). Some overexpressed proteins show some localization along the length of the dendrites, anterior to the transition zones. (G, H, I, K, L) CHE-11::GFP, OSM-5::GFP, XBX-1::GFP, KAP-1::GFP, and BBS-7::GFP are consistently mislocalized in *dyf-11* mutant animals, frequently showing protein accumulations in the dendrites and/or cilia (denoted by asterisks). The anticipated position of ciliary axonemes is noted with a dashed bracket, and transition zones with arrowheads. Question marks represent unclear localization of transition zones and cilia, or of the GFP-tagged proteins. (J) OSM-3::GFP appears to properly enter the severely truncated cilia of *dyf-11* mutants. Scale bar, 5 µm.

When combined with our finding that *dyf-11* mutant cilia are truncated to the same extent as those mutants with abrogated IFT subcomplex B components such as OSM-5/IFT88, OSM-6/IFT52, and CHE-13/IFT55-57 (Figure 2A) [55],[56], the above results further support the notion that DYF-11 associates either directly or peripherally with the IFT particle subcomplex B. Previous work has shown that OSM-6 likely anchors IFT subcomplex B at the base of the cilium, possibly at transitional fibers in proximity to the ciliary membrane [56],[62]. For example, OSM-6 can enter the cilium in *che-13* and *osm-5* mutants, but CHE-13 and OSM-5 are excluded from cilia in an *osm-6* mutant background [56]. Therefore, to further resolve the positioning of DYF-11 within the hierarchy of IFT subcomplex B, we examined the localization of DYF-11::GFP in *che-13* and *osm-6* mutant backgrounds. In both cases, DYF-11::GFP enters the truncated cilia, with little or no observable accumulation in the dendrites (Figures 4G,H). OSM-5::GFP, on the other hand, fails to enter the severely truncated cilia of *dyf-11* mutants, showing significant leakage into dendrites (Figure 5H). These data suggest that DYF-11 may be more ‘centrally’ localized (*e.g.*, closer to the OSM-3-kinesin) than OSM-5, OSM-6 and CHE-13 in the IFT particle subcomplex B, although additional studies are needed to confirm this potential protein topology.

### DYF-11 Is Required for the Integrity of the Motor-IFT Machinery

To provide additional insight into the function of DYF-11 with regards to the motor-IFT machinery and cilia formation, we analyzed by microscopy the behavior of several GFP-tagged IFT-associated components in the *dyf-11* mutant strain. In addition to the IFT subcomplex B protein OSM-5 (see above), we tested a Kinesin-II component (the Kinesin-Associated Protein 1, KAP-1), homodimeric kinesin OSM-3, a component of IFT-dynein (the light-intermediate chain XBX-1), an IFT particle subcomplex A protein (CHE-11/IFT140), and a BBS protein (BBS-7). In wild-type animals, these proteins always localize prominently at transition zones/basal bodies and clearly undergo bidirectional transport along ciliary axonemes (Figures 5A–F, and data not shown for transport rates) [31]. Remarkably, aside from OSM-3-kinesin, we reproducibly observed abnormal localization for all of these proteins in the *dyf-11* mutant strain. OSM-3::GFP consistently localizes to transition zones and the truncated amphid and phasmid cilia of *dyf-11* mutants (Figure 5J). In contrast, CHE-11, XBX-1, and BBS-7 were anchored to transition zones but their signal intensities were significantly reduced compared to wild-type, and none of the proteins were observed to enter the (truncated) amphid and phasmid cilia (Figures 5G, I, L). Interestingly, the heterotrimeric kinesin KAP-1 subunit mislocalized consistently along the dendrite, with no apparent anchoring to the transition zones and no observable localization to the ciliary axoneme (Figure 5K). All observations are reproducible and are based on the analysis of at least 50-100 animals, with the observer blind to the genotype and GFP-tagged protein under examination. It should be noted that the observed mislocalizations in the *dyf-11* mutant animals appear to be more severe than those typically seen in IFT subcomplex B mutants, perhaps hinting at additional roles for the DYF-11 protein in IFT. Overall, our findings are consistent with DYF-11 playing a critical role in the assembly and thus functions of various IFT-associated proteins, including the Kinesin-II motor, into a functional motor-IFT machinery. Whether DYF-11 operates as an integral IFT component, or potentially as an associated ‘cargo’ protein that affects the core machinery, remains to be determined.

### Vertebrate MIP-T3 Is Likely Necessary for Morphogenetic Signal Transduction

**Figure 6:**
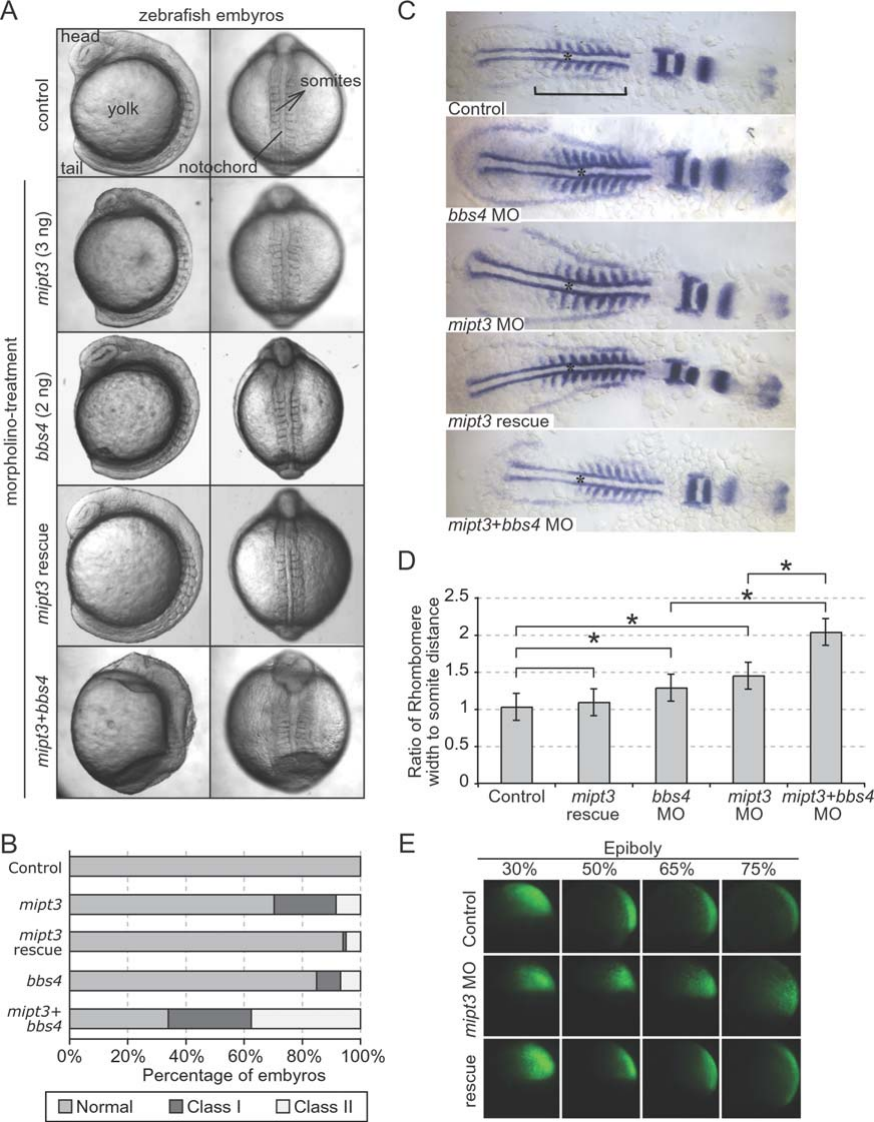
Zebrafish *mipt3* is required for morphogenetic development and acts synergistically with the gene encoding the basal body/ciliary protein, BBS4. (A) Injection of a progressively increasing amount of a translation-blocking *mipt3* morpholino (MO) gives rise to a spectrum of gastrulation phenotypes, including shortening of the embryonic axis, broadening and kinking of the notochord, lengthening of the somites and detachment of cells along the embryonic axis, all of which can be rescued with high specificity by the *mipt3* mRNA. The presence of two of these phenotypes is scored as “Class I”, whereas three or more phenotypes are categorised as “Class II”. Representative images of control, *mipt3*, *bbs4* and double morphants at the 8–10 somite stage, exhibiting a range of phenotypes. Note the profound lengthening of somites in the double morphant, as well as the loss of definition of head structures, and the complete failure of tail extension, phenotypes that are not seen in either single morphants. (B) Compared to a modest percentage of affected embryos in clutches injected with either 2 ng of a *bbs4* MO or 3 ng of a *mipt3* MO, co-injection of both *bbs4* and *mipt3* MOs leads to the widespread phenotypes mentioned in (A) that recapitulated and exacerbated the phenotypes of single morphants, with a particularly prominent expansion of Class II embryos to ∼40%. (C) Somite-stage embryos were labeled with *pax2, krox20* and *myoD* by in situ hybridization. As compared to both controls and embryos rescued by co-injecting MO with *mipt3* mRNA, morphant embryos were shorter (see bar across the somitic trunk) and exhibited wider, thinner somites. Embryos injected with both *mipt3* and *bbs4* MO were significantly shorter and wider than embryos injected with either MO alone. The notochord, which is wider upon suppression of either *mipt3* or *bbs4*, is also shown with an asterisk. (D) Quantitative assessment of morphant phenotypes. (A) The extent of gastrulation movement defects was quantified by calculating the ratio of the measurements of the width of the 5^th^ rhombomere and the distance from the rhombomere to the first somite. Morpholino injection alone revealed an increase in the ratio as compared to controls. Bars above the graph indicate comparisons; asterisks annotate statistically significant differences (p<0.05). (E) At gastrulation stages, the movements of fluorescently-labeled cells descendent of a single labeled 16-cell blastomere were tracked. At 30% epiboly, no difference in the position of cells could be observed between control embryos and morphants. As embryos proceed through epiboly, cells in control embryos converge upon the dorsal midline and extend along the dorsal axis (top panels). Cells in morphant embryos do not converge and extend, which is indicative of defective gastrulation movements (middle panels). Cell movements are rescued with co-injection of WT zebrafish *mipt3* mRNA (bottom panels). See Suppl. Fig. 4 for quantification.

To provide *in vivo* evidence that DYF-11/MIP-T3 plays a ciliary role in vertebrates, we turned to *Danio rerio* (zebrafish), whose ortholog (LOC393572) exhibits 51% amino acid identity to its human counterpart (Figure 1A). We designed a morpholino (MO) to suppress the translation of *mipt3* in zebrafish embryos, injected it at the 2-cell stage and phenotyped animals during gastrulation and somitogenesis. We and others showed recently that defects in basal body/cilia function(s) perturb non-canonical Wnt signaling, which in zebrafish manifests as a constellation of gastrulation movement defects that include shortened body axis, broad and sometimes undulated notochords, thinning and elongation of the somites, and failure of tail extension [7], [12], [63]–[65]. We found that disrupting *mipt3* phenocopied the gastrulation movement phenotypes seen with other basal body/ciliary mutants [63],[65] in a dose-dependent manner (Figures 6A 6B and Figure S3A; n = 100–150 embryos per injection) and with high specificity, since co-injection of a *mipt3* MO with a *mipt3* capped mRNA rescued >95% of the affected embryos for all phenotypes scored (n = 120; Figures 6A–E and Figure S3B and Figure S4). *mipt3* morphants were shorter than control-injected embryos at the same somitic age (9±1 somites) and had consistently broadened notochords (Class I), while more severely affected embryos (Class II) also had longer somites as observed by live embryo imaging and confirmed by *in situ* hybridization staining with riboprobes against *myod*, *pax2* and *krox20* (Figures 6C, 6D). In addition, Class II embryos also exhibited detachment of cells along the embryonic axis, probably a result of cell death whose etiology is unclear (Figure 6A), while some embryos failed to complete epiboly and/or were necrotic at the mid-somitic stage (data not shown).

To confirm that the observed defects were a result of defective gastrulation movements, we labeled a single blastomere in MO injected embryos at the 16-cell stage and followed the labeled cell populations through epiboly. As compared to control and WT rescue embryos (Figure 6E), cells converged upon the dorsal midline and extended along the axis aberrantly (Figure 6E). The observed phenotypes were quantified in somite stage embryos by a number of methods. Shortened body axes were measured via body gap angles as described previously [12]. Compared to the average control gap angle of 59°, morphant embryos exhibited a statistically significant increase of the mean angle of 86° (p<0.05; Figure S3B). Further, the broadened body and shortened body axis were quantified by an independent, objective method, where we calculated the ratio of the width of the 5^th^ rhombomere, labeled by *krox20* in situ staining, over the distance from the 5^th^ rhombomere to the first somite, labeled by *myod* staining. Once again, while morphants co-injected with zebrafish *mipt3* mRNA were indistinguishable from controls, injection of *mipt3* MO alone caused a significantly increased ratio consistent with a wider body and shortened body axis (p<0.05; Figure 6D). Finally, quantification of the efficiency of migration of cells to the midline during epiboly also showed significant differences between controls and *mipt3* morphants, a phenotype that could be rescued fully by co-injection of *mipt3* mRNA (Figure 6E and Figure S4).

Importantly, MO-based knockdown of both *mipt3* and *bbs4*, which encodes a BBS protein that localizes to centriolar satellites (proximal to basal body) and is necessary for gastrulation movements [63], led to morphogenetic phenotypes that were more severe than with the knockdown of either gene alone. Injection of subeffective doses of *mipt3* or *bbs4* MOs yielded observable phenotypes in <20% of embryos (n = 113 and 127, respectively) (Figures 6A, 6B); by contrast, co-injection of both *mipt3* and *bbs4* MOs resulted in profound defects in >60% of embryos (Figure 6B), which included not only gastrulation movement hallmarks, such as prominently broadened somites, but also gross morphological defects in severely affected embryos (Figure 6A), including decreased intraocular distance as measured by *pax2* expression (data not shown), that might hint at broader functions of *mipt3* and *bbs4*. These results were corroborated by a significant increase in both body gap angles and rhombomere width to rhombomere-somite distance ratios in *mipt3/bbs4* morphants as compared to embryos injected with either *mipt3* or *bbs4* alone (Figures 6C, 6D and Figure S3B).

### Concluding Remarks

Human MIP-T3 (Microtubule-interacting protein associated with TRAF3), also termed TRAF3IP1 (TNF Receptor-Associated Factor 3 Interacting Protein 1), is a poorly-characterized protein previously implicated in TRAF3 function and shown to bind microtubules [66]. More recently, Morris *et al* [67] found that MIP-T3 interacts with the DISC1 (Disrupted-in-schizophrenia 1) protein and is required for its localization to microtubules/centrosomes. Yet, given that MIP-T3 protein orthologs are present in organisms lacking both TRAF3 and DISC1, including *B. dendrobatidis*, *Chlamydomonas* and *C. elegans* (Figure 1A), we hypothesized that MIP-T3 likely also performs a more general, evolutionarily conserved function—one related to cilia formation [21]. Indeed, we now show that *C. elegans* MIP-T3 (DYF-11) localizes to transition zones/basal bodies and cilia, as does its mammalian counterpart (Figures 3A, B). Importantly, *C. elegans* DYF-11 is an intraflagellar transport protein critical for the formation of full-length, functional sensory cilia (Figures 1–3). Abrogating DYF-11 function produces ciliary defects consistent with a role for the protein in the IFT process itself: (i) cilia are approximately the same length as those of mutants lacking IFT subcomplex B proteins [56],[58],[68]; (ii) four representative classes of IFT-associated components—OSM-5 (IFT subcomplex B), CHE-11 (subcomplex A), XBX-1 (dynein motor subunit) and BBS-7 (part of the BBS protein complex)—fail to enter the short, residual cilium in *dyf-11* mutant animals (Figures 5G, H, I and L); (iii) remarkably, the heterotrimeric Kinesin-II motor subunit KAP-1 also mislocalizes (Figure 5K). These observations suggest that DYF-11 is not only involved in maintaining the integrity of the IFT machinery, but also may help with the assembly of Kinesin-II onto the IFT complex. Moreover, it is possible that a proportion of the *C. elegans* DYF-11/MIP-T3 protein associates directly with microtubules to influence the function of the IFT motors or components.

Another potentially pertinent discovery by Taya *et al* [69] is that the MIP-T3-interacting protein DISC1 regulates the Kinesin-1-dependent transport of a protein complex composed of NUDEL/LIS1/14-3-3ε. Consistent with a probable role in transport and neurogenesis, overexpression of a dominant-negative variant of DISC1 results in defective (shortened) neurite outgrowths [70]. Hence, one of the apparent functions of DISC1 as a cohesion factor for a multisubunit protein complex (NUDEL/LIS1/14-3-3ε) has interesting parallels to that of its interacting partner MIP-T3, which we show may function as a subunit of the IFT subcomplex B that possibly links, either directly or indirectly, a ciliary kinesin to the multiprotein IFT subcomplexes A/B (Figure 4). Whether *C. elegans* DYF-11 has dendrite-associated functions whose disruption could affect ciliogenesis represents an interesting question that will need to be addressed.

Several genome- and proteome-wide studies are in accord with our finding that MIP-T3 plays a critical role in IFT. The *Chlamydomonas* flagellar proteome uncovered by Pazour *et al* [37] identified the MIP-T3 ortholog (FAP116) as an abundant protein present specifically in the membrane-plus-matrix but *not* the axonemal fraction of cilia, precisely like other IFT proteins. *Chlamydomonas* MIP-T3 is upregulated >3.0 fold during reflagellation [39],[42], again similar to other IFT proteins and consistent with a ciliogenic role. Finally, just as in *C. elegans*, the *Drosophila* MIP-T3 ortholog (CG3259) is expressed exclusively in ciliated sensory neurons [38].

Our morpholino knockdown studies in the vertebrate *Danio rerio* (Figure 6) also suggest that MIP-T3 is likely required for basal body/ciliary functions since it is both necessary for gastrulation movements and also demonstrates a potential genetic interaction with at least one gene encoding a basal body protein (BBS4) in the regulation of this process [7],[65]. These data support the notion that vertebrate MIP-T3 plays an important role in development, potentially helping to modulate Wnt and/or other signaling pathways [12]. Intriguingly, the MIP-T3-interacting protein DISC1 binds FEZ1 [71], a protein that is implicated in neurite outgrowth and co-precipitates with BBS4 [72]. One important question raised by these observations that will need to be addressed in future studies is whether vertebrate MIP-T3 performs functions related to trafficking in neurites, possibly in cooperation with DISC1/FEZ1. If so, then such a finding would suggest that MIP-T3 may have been adapted during evolution to perform two distinct roles in protein trafficking, one in cilia as an IFT protein, and the other in neurites (axons and perhaps also dendrites).

## Materials and Methods

### Strain Construction and Maintenance

All *C. elegans* strains were maintained at 20°C, and standard genetic crosses were employed to introduce GFP reporter constructs (transcriptional or translational) into wild-type (N2) or mutant animals. PCR or dye-filling assays were used to follow genotypes, as described [36]. The following mutant strains were used in this study: *bbs-1*(*ok1111*), *bbs-7/osm-12*(*n1606*), *bbs-8*(*nx77*), *che-3*(*e1124*) *che-11*(*e1810*), *che-13*(*e1805*), *daf-2*(*e1370*), *daf-16*(*mu86*), *dyf-11*(*mn392*) *klp-11*(*tm324*), *osm-3*(*p802*), *osm-5*(*p813*), and *osm-6*(*p811*).

### Construction of Strains Harboring a Translational DYF-11::GFP Construct

A translational DYF-11::GFP fusion construct was made by fusion PCR as described [53]. The entire genomic coding region of *dyf-11* (C02H7.1), along with 528 bp of promoter sequence 5′ of the start codon, was fused upstream of, and in frame with, the GFP coding sequence. Aside from DYF-11::GFP, the following strains were used: *dpy-5*(*e907*); Ex[*gcy-5p*::gfp+*dpy-5*(+)] *dpy-5*(*e907*); nxEx[*osm-12*::gfp+*dpy-5*(+)], N2; myEx10[*che-11::gfp*+*rol-6*(*su1006*)]; N2; Ex[*kap-1::gfp*+*rol-6*(*su1006*)], N2; ejEx1[*osm-3::gfp*+*rol-6*(*su1006*)], N2; yhEx2[*osm-5::gfp*+*rol-6*(*su1006*)], N2; Ex[*srb-6p*::gfp+*rol-6*(*su1006*)] and N2; nxEx[*xbx-1::gfp*+*rol-6*(*su1006*)].

### Localization of MIP-T3 in Mammalian Cells

A tagged expression construct for MIP-T3 was generated by LR clonase II (Invitrogen) mediated recombination between the pENTR 221-MIP-T3 (Ultimate ORF clone IOH28851; Invitrogen) and pcDNA6.2/nLumio-DEST (Invitrogen), placing the human MIP-T3 ORF under control of a CMV promoter with an N-terminal V5 epitope tag (pcDNA6.2/nLumio-MIP-T3).

IMCD3 cells were plated on glass coverslips and transfected with the pcDNA6.2/nLumio-MIP-T3 vector when cells reached 60% confluency by using FuGENE6 (Roche) transfection reagent. Twenty-four hours post-transfection, cells were fixed in methanol and stained using mouse anti-V5 (1∶200, Invitrogen), mouse anti- γ-tubulin or mouse anti- acetylated-tubulin (both 1∶1000, Sigma), and secondary detection carried out with goat anti-mouse IgG antibody conjugated to Alexa 488 dye, and goat anti-mouse IgG antibody conjugated to Alexa 594 (both 1∶1000, Invitrogen). Cells were visualized by fluorescence microscopy.

### Cloning of dyf-11 (C02H7.1)

The *C. elegans* MIP-T3 gene homolog, C02H7.1, is physically situated close to the interval defined for the *dyf-11(mn392)* mutant allele (X: −18.27±0.244 cM) [43], suggesting that the genetic lesion lies within this gene. We sequenced the C02H7.1 coding region in the *dyf-11* mutant and uncovered a nonsense mutation (TCA→TGA) in the third exon at nt 419 of the coding region. Primer sets used to detect the mutation were: OPS0320 TGGTCGCAATTTGACCACC and OPS0322 TGATCATTCTCGGGCTCTC (fragment 1); OPS0321 GACGATCATGAGATTTCTG and OPS0323 CAACATATTGGTGCAACTTC (fragment 2). A second putative *dyf-11* allele, *ad1303*, had no sequence alterations in exons and complemented the *dyf-11(mn392)* mutation, suggesting that it represents a different gene.

### *C. elegans* Phenotypic Analyses

Dye-filling assays using the fluorescent dye DiI were performed as described [53]. Chemotaxis assays were carried out for 1 hour essentially as described [46], using isoamyl-alcohol as a chemoattractant. A chemotaxis index was calculated as the number of worms in attractant zone minus worms in control zone, divided by the total number of worms. Osmo-avoidance assays were performed as described [46]. Briefly, ∼5 worms (for each of at least 20 assays) were placed inside a small ring of 8 M glycerol, and animals found within or beyond the ring after 10 minutes were counted as non-avoiders. An osmo-avoidance index was calculated: (total avoiders–non-avoiders)/total worms.

Lifespan assays were based on the protocol of Apfeld and Kenyon [49]. Animals were grown for at least one generation at 20°C before eggs were collected. At the L4 molt, worms were transferred to NGM plates containing 16 µM fluorodeoxyuridine (FUDR) to prevent progeny growth and kept at 20°C throughout the assay. 100 worms were picked for each strain, at 10 worms/plate. Worms were scored every 1–2 days for viability; those no longer responding to prodding with platinum wire were considered dead, and those that exploded or crawled off the plate were censored.

To test for entry into and exit from the dauer stage, we employed an existing strategy [73]. 10 adult worms were allowed to lay eggs on plates with food at 20°C for 4 hours. Adults were then removed and eggs counted. Eggs were allowed to develop for 4 days at 20°C or 3 days at 25°C, after which they were scored as either Daf-c or Daf-d as follows. To identify Daf-c worms, plates were flooded with 1% SDS, where only dauer larvae remained as live thrashing animals after 15 minutes. To identify Daf-d worms, animals were allowed to grow 4 days following complete consumption of food, after which they were exposed to and unable to survive the 1% SDS treatment. *daf-16* (*mu86*) and *daf-2* (*e1370*) were used as Daf-d and Daf-c controls, respectively. All dauer/lifespan assays were carried out in duplicate or triplicate.

Nile Red staining was performed as previously described [50]. Briefly, Nile Red powder (Molecular Probes) was dissolved in acetone as a 1mg/ml stock solution and kept at room temperature. The stock solution was diluted in 1x PBS to 1 ug/ml and 0.5 ml of diluted solution was applied to NGM plates seeded with *E.coli* OP50. Plates were allowed to dry for 24 hours. Staged eggs were allowed to develop on Nile Red plates at 20°C. Worms were transferred every 2 days to fresh Nile Red plates and were analyzed two days post-L4 by fluorescence microscopy. Images were captured and processed under identical settings using OpenLab software (Improvision, Inc). Nile Red fluorescence intensity was calculated as the mean pixel intensity after background subtraction.

### Analysis of Sensory Neuron Structure and Cilia Length Measurements

ASER amphid and PHA/PHB phasmid sensory neuron structures were visualized by expressing the cell-specific transcriptional reporters, *gcy-5p::gfp* and *srb-6p::gfp* [36], respectively. In these neurons, the GFP diffuses freely throughout the neuron to mark cell bodies, axons, dendrites, transition zones and cilia. Cilium length was measured from the distal end of the transition zone (visible as a ‘bulge’ of fluorescence) to the distal tip of the cilium.

### Visualization of IFT and Rate Measurements by Time-Lapse Microscopy

Transgenic animals expressing GFP-tagged proteins were mounted on agarose pads and immobilized with 20 mM levamisole. Amphid or phasmid cilia were examined with a 100X, 1.35 NA objective and an ORCA AG CCD camera mounted on an Zeiss Axioskop 2 mot plus microscope. Images and movies were obtained in Openlab version 5.02 beta (Improvision). Kymographs were generated using the MultipleKymograph ImageJ plug-in. Rates from middle and distal segments were obtained essentially as described in Snow *et al* [54].

Images for intraflagellar transport were collected using a Zeiss Axiovert 200 equipped with a Hamamatsu Orca AG CCD camera, spinning disk confocal head, Zeiss Plan-neofluar 63X, 1.3 NA, water-immersion objective and a 1.5X magnification lens Improvision Piezo Focus Drive. Images for MX488 *bbs-7(n1606); Ex[dyf-11::GFP+dpy-1(+)]* and *MX486 N2; Is[dyf-11::GFP+dpy-1(+)]* were collected at 7.5 frames/sec and 4.24 frames/sec respectively. Animals were first anaesthetized with 10 mM levamisole, mounted on agar pads and photobleached for 300–1900 ms before images were collected for 2 minutes. The FRAP module is a Photonic Instruments MOSAIC Digital Diaphragm System with a 488 nm 300 mW laser line. Images were collected using Volocity.

### Zebrafish Morpholino Knockdown Studies

We obtained a translational blocking *mipt3* morpholino (MO) from Gene Tools Inc (5′- ACCGATTCGTTCATGGCATCAAACC-3′). The MO was diluted to the desired concentrations in deionized, sterile water and injected into 2-cell stage embryos as described [64]. To rescue the morphant phenotypes, we amplified the open reading frame of zebrafish *mipt3* and cloned it into the pCS2+ vector, from which we transcribed RNA using the SP6 mMessage mMachine kit (Ambion). Phenotypes and imaging were performed as described [64].

In situ hybridization, monitoring of gastrulation movements, and measuring of embryo body gap angles were carried out using previously described methods [12]. Measurements of rhombomere width and rhombomere-somite distances were taken on flat-mounted embryos photographed at 10X magnification. Intraocular distance was taken as the measurement of the width of the *pax2-*expressing region, labeled by ribostaining, and was measured on flat-mounted images photographed at 10X. All experiments were performed blinded to the injection cocktail.

## Supporting Information

Movie S1 Time-lapse microscopy (4 frames/second) of *C. elegans* DYF 11::GFP seen moving bi-directionally along amphid cilia, as with other IFT proteins.
(0.56 MB MOV)

Figure S1 Amino acid alignment of *C. elegans* DYF-11 (C02H7.1) with MIP T3 protein orthologs from *C. reinhardtii* and *H. sapiens*. Identical and similar residues are highlighted in black and gray, respectively. Note that the major difference in size between the human and *C. elegans/Chlamydomonas* proteins are due to two large insertions found in the human protein sequence. The number of residues is displayed at the ends of the sequences. *Caenorhabditis elegans* DYF-11/MIP-T3: C02H7.1; *Homo sapiens* MIP-T3: AAF76984; *Chlamydomonas reinhardtii* MIP-T3 accession number: C_140070.
(1.59 MB EPS)

Figure S2 Fluorescence images indicating the possible presence of DYF 11::GFP in the distal segments of AWC neuron cilia. Two of the highlighted extensions (hollow arrowheads) from the DYF-11::GFP protein overlap with the RFP protein that is most highly expressed in AWC cilia. Two ‘branches’ of the AWC cilium are shown with arrows, and the bundle of amphid channel cilia are pointed to. The schematic shows the ultrastructure of the AWC cilium, as visualised in Perkins et al. (1986). Images were acquired in the strain OE3657 *dpy-5(e907)* I; *dyf-11(mn392)* X; nxEx[C02H7.1::gfp; *dpy-5* (+)]; ofEx457 [*odr-3*::rfp; *elt-2*::cherry].
(4.94 MB TIF)

Figure S3 Gastrulation phenotypes in mipt3 morphant embryos. (A) Injection of a progressively increasing amount of a translation-blocking mipt3 morpholino (MO) gives rise to a spectrum of gastrulation phenotypes, including shortening of the embryonic axis, broadening and kinking of the notochord, lengthening of the somites and detachment of cells along the embryonic axis. The presence of two of these phenotypes is scored as “Class I”, whereas three or more phenotypes are categorized as “Class II”. (B) Body gap angle measurements for *mipt3* and *bbs4* morphants. The gap angle of mid-somitic embryos (nine somites +/− one somite) as defined by the angle formed by triangulating three points (tip of head, tip of tail, center of yolk; see also Gerdes et al., 2007) was calculated to capture the mean length of embryo populations (n = 50–70 embryos). On the y-axis, the angle is plotted (in degrees) while the x-axis shows the various injection cocktails. The phenotype is rescued efficiently by co-injection of capped *mipt3* mRNA. Note the significantly shorter embryos in the *mipt3*+*bbs4* double morphants. Data were calculated blind to injection cocktail; bars depict standard error.
(9.82 MB TIF)

Figure S4 Quantification of gastrulation movement defects during epiboly. The mean width of fluorescein-positive region was measured across each time-point assayed in nine embryos per category (control, *mipt3* morphant, and *mipt3* rescue). Asterisks indicate statistically significant differences (p<0.05) between morphants and controls or rescued embryos; the latter two were indistinguishable from each other.
(0.47 MB TIF)
