# Supplementary figures and images for: An Essential Role for DYF-11/MIP-T3 in Assembling Functional Intraflagellar Transport Complexes
Source: PLoS Genet. 2008 Mar 28;4(3):e1000044. doi: 10.1371/journal.pgen.1000044 (PMC2268012; doi:10.1371/journal.pgen.1000044)

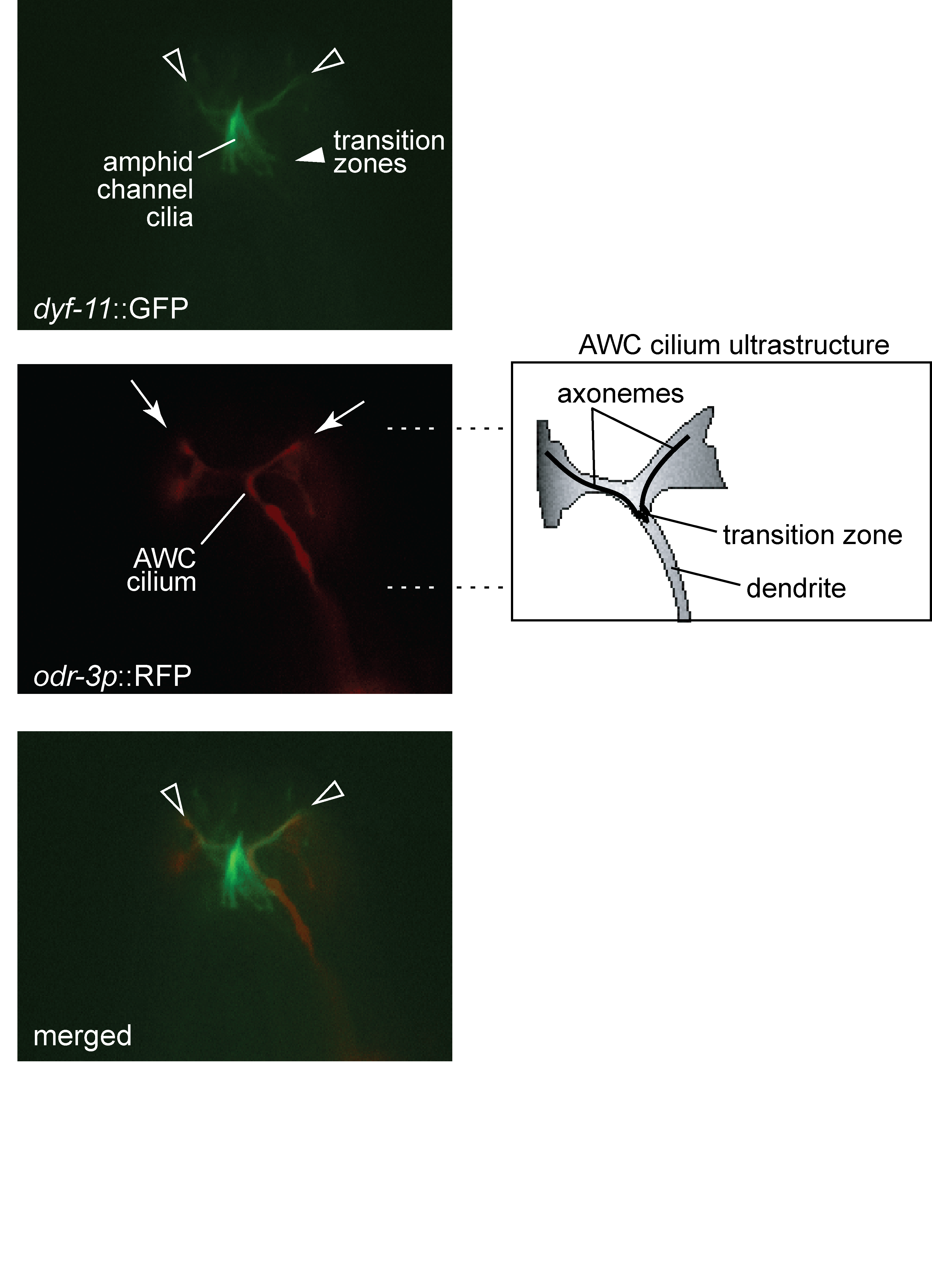

Supplement: Figure S2 — Fluorescence images indicating the possible presence of DYF 11::GFP in the distal segments of AWC neuron cilia. Two of the highlighted extensions (hollow arrowheads) from the DYF-11::GFP protein overlap with the RFP protein that is most highly expressed in AWC cilia. Two ‘branches’ of the AWC cilium are shown with arrows, and the bundle of amphid channel cilia are pointed to. The schematic shows the ultrastructure of the AWC cilium, as visualised in Perkins et al. (1986). Images were acquired in the strain OE3657 dpy-5(e907) I; dyf-11(mn392) X; nxEx[C02H7.1::gfp; dpy-5 (+)]; ofEx457 [odr-3::rfp; elt-2::cherry]. (4.94 MB TIF) [file pgen.1000044.s003.tif]

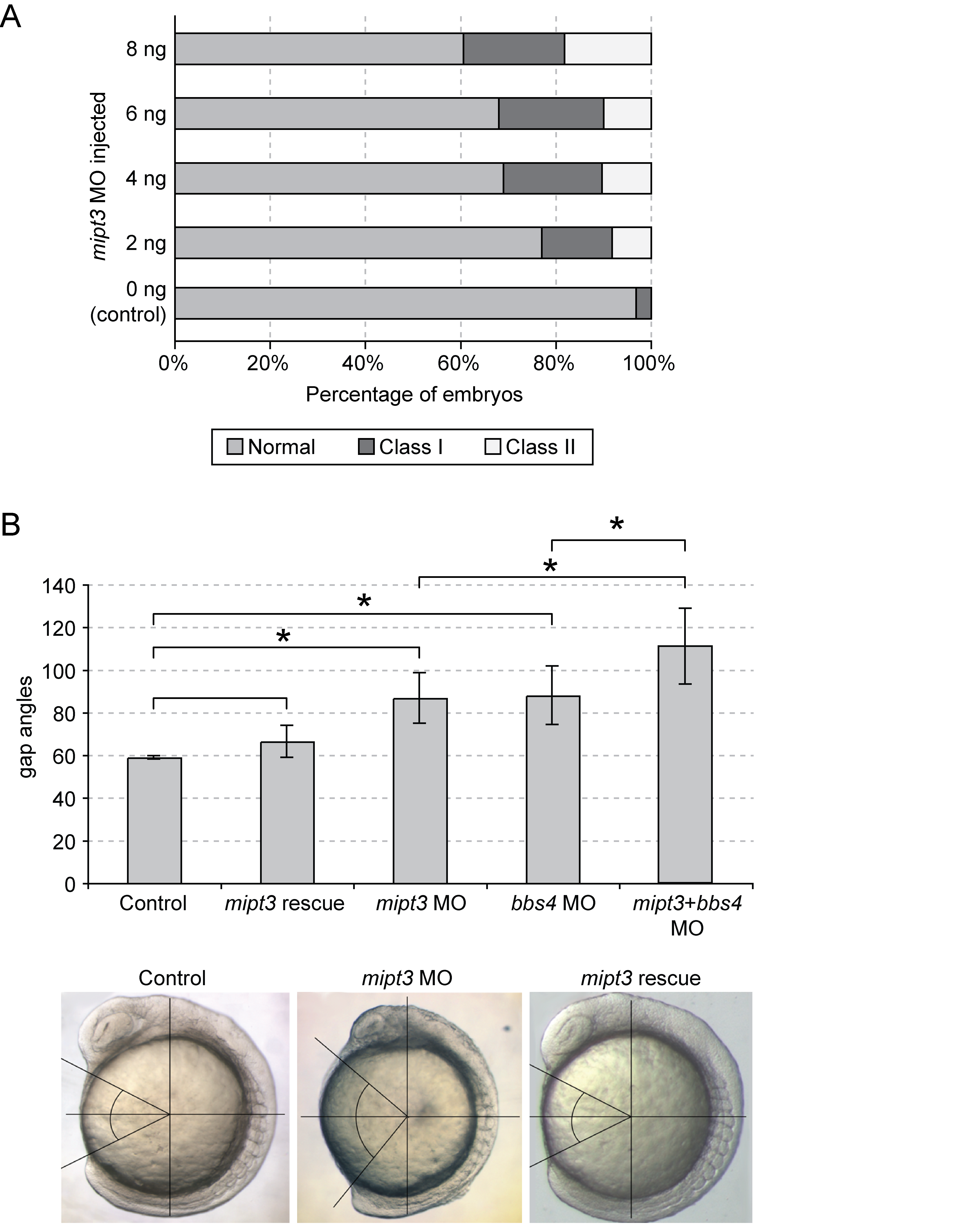

Supplement: Figure S3 — Gastrulation phenotypes in mipt3 morphant embryos. (A) Injection of a progressively increasing amount of a translation-blocking mipt3 morpholino (MO) gives rise to a spectrum of gastrulation phenotypes, including shortening of the embryonic axis, broadening and kinking of the notochord, lengthening of the somites and detachment of cells along the embryonic axis. The presence of two of these phenotypes is scored as “Class I”, whereas three or more phenotypes are categorized as “Class II”. (B) Body gap angle measurements for mipt3 and bbs4 morphants. The gap angle of mid-somitic embryos (nine somites +/− one somite) as defined by the angle formed by triangulating three points (tip of head, tip of tail, center of yolk; see also Gerdes et al., 2007) was calculated to capture the mean length of embryo populations (n = 50–70 embryos). On the y-axis, the angle is plotted (in degrees) while the x-axis shows the various injection cocktails. The phenotype is rescued efficiently by co-injection of capped mipt3 mRNA. Note the significantly shorter embryos in the mipt3+bbs4 double morphants. Data were calculated blind to injection cocktail; bars depict standard error. (9.82 MB TIF) [file pgen.1000044.s004.tif]

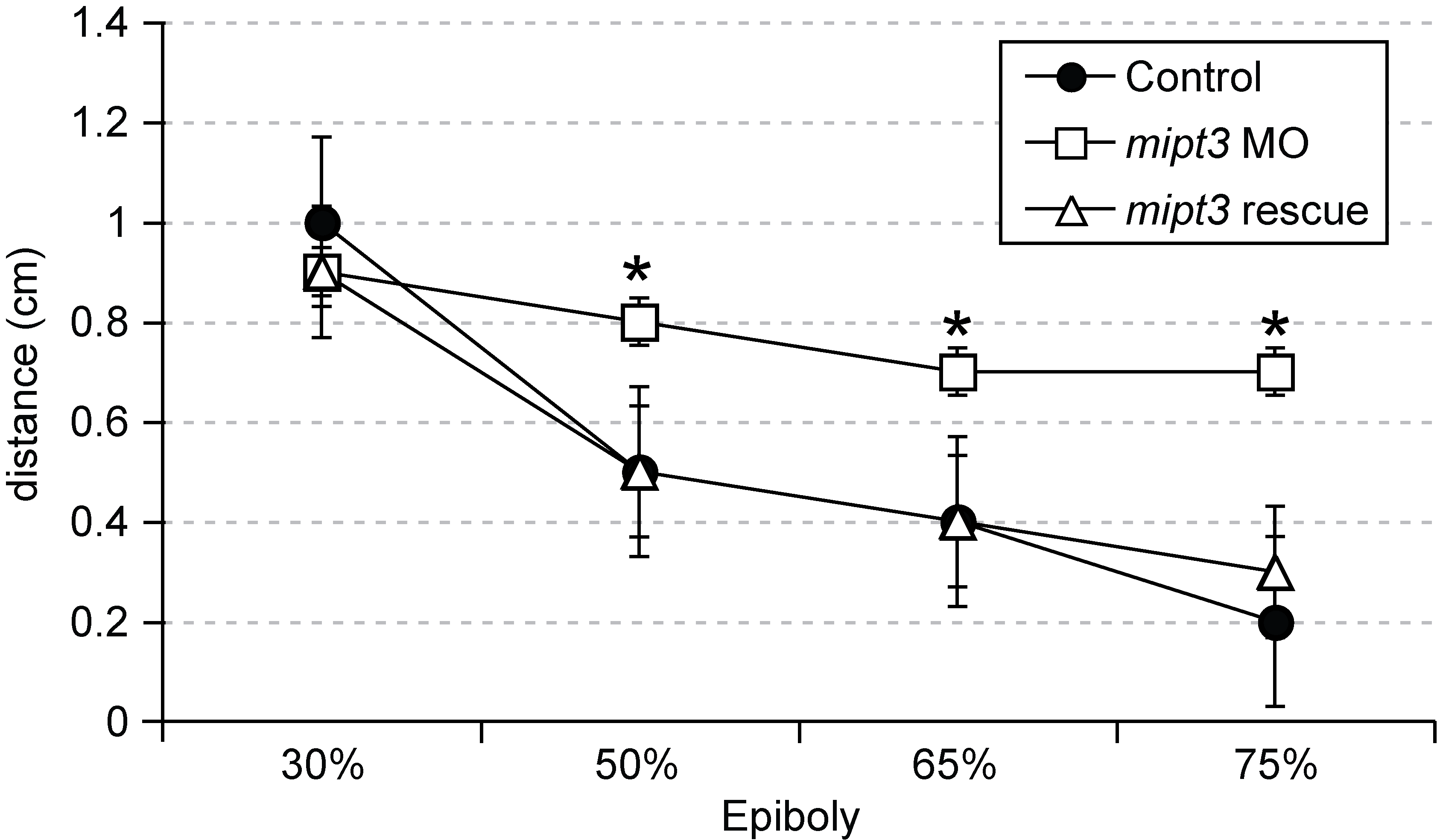

Supplement: Figure S4 — Quantification of gastrulation movement defects during epiboly. The mean width of fluorescein-positive region was measured across each time-point assayed in nine embryos per category (control, mipt3 morphant, and mipt3 rescue). Asterisks indicate statistically significant differences (p<0.05) between morphants and controls or rescued embryos; the latter two were indistinguishable from each other. (0.47 MB TIF) [file pgen.1000044.s005.tif]
